# Characterization of Volatile Organic Compounds and Aroma Sensory Properties in Yunnan Cigar

**DOI:** 10.1155/2024/9583022

**Published:** 2024-10-16

**Authors:** Yuping Wu, Haiyu Zhang, Wenyuan Wang, Guanghui Kong, Zaiming Li, Tikun Zhang, Miaochang Wang, Dong Yang, Chengming Zhang, Yongping Li, Jin Wang

**Affiliations:** ^1^Yunnan Academy of Tobacco Agricultural Science, Yuxi, Yunnan 653100, China; ^2^College of Chemical and Environment, Yunnan Minzu University, Kunming 650500, China; ^3^Research and Development Center, China Tobacco Yunnan Industrial Co., Ltd, Kunming 650231, China; ^4^Puer Branch of Yunnan Tobacco Company, Puer 665099, Yunnan, China

## Abstract

To characterize volatile organic compounds (VOCs) and aromatic sensory properties in Yun cigar, 27 samples from four origins were analyzed using SPME–HS–GC/MS and sensory analysis. The investigation results were analyzed using principal component analysis (PCA), Fisher linear discriminant analysis (LDA), and Pearson correlation analysis. In Yunnan cigars, the content of nicotine and neophytadiene accounted for over 90% of the total VOC content. Nicotine was significantly positively correlated with neophytadiene and phytol. The cigars from four origins were clearly classified by the PCA of VOCs. Four region discrimination functions were established through the LDA of 14 compounds, and the validation accuracy was 100%. The sensory descriptors with the highest geometric mean were woody, roasted, fresh-sweet, bean, and scorched. Acetophenone, megastigmatrienone A, and thunbergene were positively correlated with multiple aroma descriptors, while nicotine was negatively correlated with multiple aroma descriptors.

## 1. Introduction

Cigar is a high-end tobacco product, made from dried and fermented tobacco leaves [1]. The cigar has a unique aroma and taste and is widely favored by consumers. With the improvement of the economic level, the consumption of cigars has grown rapidly [2, 3]. At present, the global market scale of cigars has reached 20 billion US dollars [4, 5]. China has also increased the cultivation and production of cigar tobacco. Yunnan, as the main tobacco origin in China, has gradually increased cigar tobacco cultivation. The main cigar-producing areas in Yunnan, including Yuxi, Lincang, Pu'er, and Dehong, have achieved a cigar tobacco cultivation exceeding 70,000 acres, accounting for more than 50% of China's. However, there is little research on the aroma and flavor of Yunnan cigars.

Characteristics: the aroma of Yunnan cigars is beneficial for its product positioning and marketing. Volatile organic compounds (VOCs) are the material basis of tobacco aroma and play an essential role in its quality [6, 7]. There are over 500 VOCs in tobacco, which are mainly influenced by tobacco variety, origin, and processing technology [8–11]. For instance, Cuban cigars are renowned for their unique blend of leather, cedar, spice, cocoa, dark fruit, earth, and pepper flavors, attributed to the specific terroir and curing techniques [12]. The content of VOCs such as benzaldehyde, phytol, megastigmatrienone B, and neophytadiene in Cuban cigar tobacco leaves is relatively high [12]. Similarly, Indonesian cigars smell molasses with dried fruit, leathery, peppery, and baked flavor, and its prominent VOCs were beta-ionone, damascone, solanone, benzaldehyde, and cedrol [13].

The VOCs are mainly analyzed by gas chromatography–mass spectrometry (GC/MS). Solid phase microextraction (SPME) technology is a fast and efficient pretreatment method that integrates sampling, extraction, concentration, and injection [14]. The combination of SPME and GC/MS has been widely used to detect VOCs in agricultural products such as rice, tea, wine, and honey [7, 15, 16].

In addition, sensory analysis is the qualitative and quantitative description and evaluation of products by trained evaluators [17, 18]. Sensory aroma analysis technology is currently the primary method for evaluating tobacco quality, and it can also be used to provide a comprehensive sensory evaluation of products such as cigars.

Another main direction of flavor research is elucidating the interrelationships between sensory and chemical components [17, 18]. Although there are hundreds of VOCs in tobacco, only a small portion of them impact its flavor [19–21]. These compounds exhibit the rich flavors of cigars through specific ratios or combinations [20, 21].

This paper aims to identify characteristics of the VOCs and aroma sensory in Yunnan cigars through SPME–HS–GC/MS and sensory evaluation. We identify Yunnan cigars through principal component analysis (PCA) and linear discriminant analysis (LDA). The correlation between inner VOCs and the correlation between VOCs and aroma are also studied by Pearson correlation analysis.

## 2. Materials and Methods

### 2.1. Samples

The samples were collected from cigar tobacco leaves from four production areas in Yunnan, including Yuxi (YX, *n* = 8), Lincang (LC, *n* = 8), Pu'er (PE, *n* = 7), and Dehong (DH, *n* = 4) in 2021 and 2022. A portion of the cigar tobacco leaves are rolled into cigars for sensory evaluation, while a portion of the cigar leaves are ground for GC/MS analysis.

### 2.2. Instruments and Reagents

GC/MS (890B–5977B) was utilized, manufactured by Agilent Technologies, USA. The DB-5MS capillary chromatography column (60 m × 250 *μ*m × 0.25 *μ*m) was also from Agilent Technologies, USA. The extraction head (50/30, DVB/CAR/PDMS) was obtained from Superco, USA, and the MPS multifunctional sampler was provided by Gerstel, Germany.

All reagents were of analytical grade and purchased from Aladdin Reagent Company and directly used for testing.

### 2.3. SPME–HS–GC/MS Analysis

About 0.2 g of tobacco powder (accurate to 0.01 g) was accurately weighed and placed in a 20-mL brown headspace vial. About 5 *μ*L of 0.5 *μ*g/*μ*L deuterated toluene *n*-hexane solution was added as the internal standard, and the vial cap was quickly tightened for analysis using SPME–HS–GC/MS.

SPME–HS conditions: The injection volume is 10 *μ*L. The equilibrium time is 5 min, the extraction temperature is 80°C, the extraction time is 20 min, and the desorption time is 5 min.

GC conditions: Helium is used as the carrier gas, with a flow rate of 1 mL/min and a split ratio of 5 : 1. The sample is automatically injected. The temperature programming is as follows: Start at 40°C, rise at a rate of 5°C/min to 230°C, and hold for 10 min.

MS conditions: The ion source temperature is 230°C, and the transmission line temperature is 270°C. MS was performed under 70 eV of electron energy and analyzed within a scanning range of 40–350 amu with a solvent delay of 8.5 min.

### 2.4. Sensory Analysis

The cigar aroma sensory evaluation was conducted by 6 professional cigarette evaluators after training. The references of aroma descriptors used in evaluators training are shown in Table S1. According to the standards YC/T 530-2015 [22], the evaluators qualitatively described and quantitatively scored the aroma of cigars. Each aroma description was scored on a scale of 0–5 based on the strength of the aroma. Then, according to ISO 11035 [23], the frequency, intensity, and geometric mean (GM) of each aroma description were calculated to evaluate the aroma characteristics of the cigar.

### 2.5. Data Analysis

Analysis of variance (ANOVA) was performed using IBM SPSS Statistics 26.0, with the *P* value set to 0.05. Fisher's stepwise LDA was also conducted by IBM SPSS Statistics 26.0. PCA was performed by SIMCA 14.1. Pearson correlation analysis was performed by Origin 2021.

## 3. Results and Discussion

### 3.1. VOCs in Yunnan Cigars From Different Origins

For VOCs qualitative, retention indices were calculated after analyzing the C7-C20 *n*-alkane series under the same chromatographic experimental conditions. Compound identification was conducted based on the MS matching of the MS database (NIST library 2017) and the retention indices. For quantitative, the internal standard deuterated toluene is used to calculate the concentration of each VOC, and the results are shown in Table 1.

These VOCs include 3 acids, 3 esters, 3 aldehydes, 19 ketones, 5 alcohols, 8 hydrocarbons, 11 alkaloids, 6 heterocyclics, and three others. There are 16 common compounds in cigars from four origins, including nicotine, myosming, anatabine, cotinine, 2,3′-dipyridyl, 1-(3-pyridinyl)-ethanone, 1-methyl-2-pyrrolidinone, neophytadiene, megastigmatrienone A (mega A), megastigmatrienone B (mega B), 3-oxo-*α*-ionol, benzaldehyde, 6-ethyl-5,6-dihydro-2H-pyran-2-one (pyran), 3-(4,8,12-trimethyltridecyl)furan (furan), phytol, and 1,1′-[1,2-ethanediylbis(oxy)] bis-benzene.

It can be found that the nicotine content is the highest, followed by neophytadiene, which accounts for more than 90% of the total VOC content in cigar tobacco. Alkaloids, especially nicotine, are the main substances that produce physiological satisfaction in tobacco [26]. PE cigar has the highest nicotine content and LC has the lowest. Free-state nicotine has irritancy when smoking, while acidic substances can form nicotine salts with nicotine, reducing its irritancy [27, 28]. Therefore, acid can, to some extent, reduce the irritancy of tobacco. PE cigar has the highest acid content and the lowest DH content. Neophytadiene is a chlorophyll degradation product and also the main VOC in tobacco [29]. Meanwhile, neophytadiene has a delicate aroma, which helps to increase the sweetness and reduce the irritancy of tobacco [29, 30]. The same as nicotine content, PE cigar has the highest neophytadiene content, and LC has the lowest. Ketones are important aroma compounds. In Yunnan cigar tobacco, we have detected various ketone aroma compounds such as megastigmatrienones, ionone derivatives (floral) [31], geranylacetone (fruit-like) [32], farnesylacetone (sweet and green) [33], and acetophenone (sweet and orange aroma) [34]. Megastigmatrienone has fruit and tobacco flavors, and we detected three of its five isomers [35, 36]. DH cigars have the highest ketone content, and YX has the lowest. Heterocyclic compounds such as pyrazine and furan are flavor compounds produced by the tobacco Maillard reaction [37], with the highest content in PE and the lowest content in LC cigar tobacco leaves.

### 3.2. PCA and Discrimination of Yunnan Cigars From Different Origins

**Figure 1:**
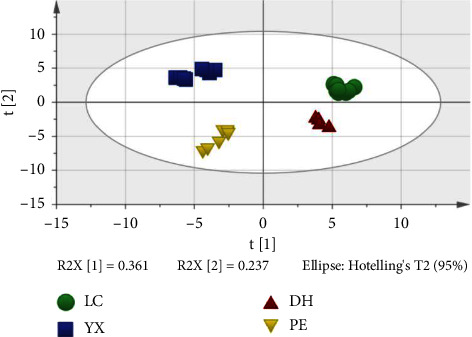
The PCA of cigars using HS–SPME–GC–MS data.

To distinguish cigars from different regions, the data of 62 compounds detected were imported into SIMCA 14.1 software for PCA. The results are shown in Figure 1. It can be seen that there is a clear boundary between cigars from different origins, and there is no sample crossing, which can effectively distinguish cigars from different origins. It indicated that Yunnan cigars from four origins can be traced through their VOCs.

Furthermore, Fisher's stepwise LDA established traceability models for four origins. In Fisher's stepwise LDA algorithm, project the data onto a low dimensional space and obtain the LDA discriminative classification function by maximizing the between-class scatter and minimizing the within-class scatter [38]. Thirteen characteristic chemical components, including pyrazine, methyl-, 5-hepten-2-one, 6-methyl-, 2,5-furandione, 3,4-dimethyl-, and acetophenone, were selected for modeling and constructing discriminant functions for the origin of cigars from four different regions, and the results are shown in Table 2. The origin discrimination function was validated, and the results are shown in Table 3. In the initial validation and leave-one-out cross-validation results, cigars from different regions were correctly classified, with the validation accuracy of 100%. Therefore, based on Fisher's LDA, cigars from 4 regions can be identified, and the selected key chemical components are the key components for their production regions.

### 3.3. Correlation Between VOCs in Yunnan Cigar

**Figure 2:**
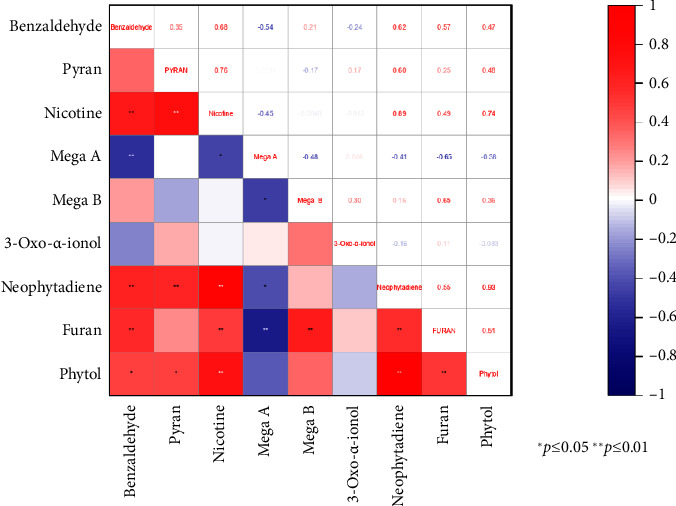
The correlation of the VOCs in Yunnan cigar.

VOCs are the main components that contribute to the flavor of food, and their presence and interaction directly affect the overall flavor of food. By studying the correlation between volatile compounds, we can better understand the mutual influence and synergistic effects between different compounds, thereby revealing the mechanisms and laws of flavor formation. To study the intrinsic relationship between main VOCs, we selected 9 out of 16 common VOCs for Pearson correlation analysis based on their content (> 50 mg/kg) and aroma (odor activity value, namely, OAV is equal to the sample concentration divided by the odor threshold, OAV > 1), and the results are shown in Figure 2.

From Figure 2, it can be seen that there is a significant positive correlation between phytol and neophytadiene, with a correlation coefficient of 0.93. This may be due to the degradation of chlorophyll to obtain phytol, and the dehydration of phytol to obtain neophytadiene, both of which belong to the chlorophyll degradation metabolic pathway [29, 30]. The correlation coefficients between nicotine, neophytadiene, and phytol are 0.89 and 0.74, respectively. This may be because nicotine and chlorophyll both belong to the N metabolic pathway [39]. Mega A and mega B are two isotopes of megastigmatrienone [35, 36], so their correlation coefficient is −0.48, indicating a significant negative correlation. The correlation coefficients between nicotine and pyran and furan were 0.78 and 0.48, respectively, indicating a significant positive correlation. Pyran and furan are products of the Maillard reaction between amino acids (proteins) and sugars [37]. Amino acids (proteins) and nicotine both belong to the N metabolic pathway; therefore, they are significantly positively correlated.

### 3.4. Sensory Analysis of Yunnan Cigars

A sensory analysis was conducted on the odor of 27 cigars from four production areas, and 27 aroma descriptions were obtained, including 21 aromas (nut, bean, coffee, cocoa, woody, spice, fruity, fresh-sweet (F-sweet), burnt-sweet (B-sweet), honey-sweet (H-sweet), floral, incense, medicinal, creamy, resinous, roasted, hay, leather, pepper, Earth, and ester) and 6 offensive odors (protein, metallic, green, scorched, pollen, and ligneous).  Table 4 shows the frequency, intensity, and GM results of different descriptors of Yunnan cigars. It can be observed that the most frequent descriptions are woody, F-sweet, and Earth. The strongest intensity descriptions are woody, roasted, and scorched. The square root of the frequency and intensity is the GM value, while sensory descriptions of GM greater than 50% include woody, roasted, F-sweet, bean, and scorched. These 5 sensory descriptions can be considered as the characteristic aroma of Yunnan cigars.

### 3.5. Correlation Analysis Between Aroma and VOCs in Yunnan Cigars

**Figure 3:**
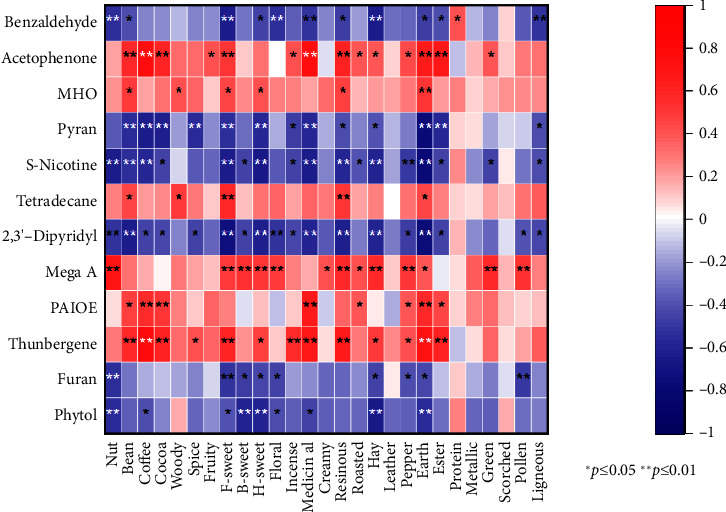
The correlation between partial VOCs and aroma descriptions of Yunnan cigar (6-methyl-3,5-heptadiene-2-one [MHO]; phthalic acid, isobutyl octyl ester [PAIOE]).

The Pearson correlation analysis between the aroma of Yunnan cigars and VOCs is shown in Table 4 and S2, and Figure 3. As the characteristic aroma of Yunnan cigars, from Table 4 and Figure 3, it can be found that woody is significantly positively correlated with tetradecane and 6-methyl-3,5-heptadiene-2-one, with correlation coefficients of 0.481 and 0.407, respectively. Tetradecane is a vital odor cue of plant pests [40, 41], with mild wax flavor. 6-Methyl-3,5-heptadiene-2-one is usually detected in tea, with fruity, sweet, and woody odor descriptions [42, 43]. The woody aroma of Yunnan cigar may be caused by 6-methyl-3,5-heptadiene-2-one and tetradecane, and there may also be other possibilities due to the complexity of the senses. Roasted is significantly positively correlated with mega A and phthalic acid, isobutyl octyl ester, with correlation coefficients of 0.416 and 0.399, respectively. It is significantly negatively correlated with S-nicotine, with a correlation coefficient of −0.395. Mega A has a tobacco aroma and a spicy aroma [25]. Phthalic acid, isobutyl octyl ester is detected in various plants [44], and it has no odor. It is commonly used as a plasticizer and additive [44]. The roasted aroma of cigar may mainly come from mega A, and phthalic acid, isobutyl octyl ester may play a coordinating role, or phthalic acid, isobutyl octyl ester's decomposition products may enhance the roasted aroma. F-sweet is significantly correlated with multiple components, among which it is negatively correlated with 2,3′-dipyridyl and benzaldehyde, with correlation coefficients of −0.722 and-0.632, and positively correlated with thunbergene and tetradecane, with correlation coefficients of 0.597 and 0.570, respectively. Thunbergene can be decomposed into solanone with sweet carrot odor [45] and diketone with buttery-caramel flavor [46]. The cigars' F-sweet flavor may mainly come from thunbergene, while the bitter almond flavor of benzaldehyde [47] may have a masking effect on it. Bean is positively correlated with multiple components such as acetophenone and thunbergene, with correlation coefficients of 0.579 and 0.568, respectively. It negatively correlates with multiple components such as 2,3 ′-dipyridyl (−0.629) and pyran (−0.557). Pyran can be detected in fungal metabolites [48], with vanilla aroma and slight bitterness. The bean flavor may mainly come from the degradation products of thunbergene and acetophenone with floral fragrance, while pyran has an inhibitory effect on it. It is to be noted that VOCs significantly correlated with scorched. Therefore, it is possible to highlight the aroma style of Yunnan cigars by regulating these VOCs.

From Table S2, acetophenone is significantly positively correlated with 14 factors, including medicinal, coffee, ester, Earth, resinous, cocoa, bean, F-sweet, pepper, fruity, incense, green, burnt, and hay. Acetophenone has a mild and aromatic odor [34]. Its fragrance is described as floral fragrance, almond-like, and hawthorn-like. All kinds of perfumes with the smell of honeysuckle, jasmine, hawthorn, or new hay contain acetophenone [34]. Mega A is significantly positively correlated with 13 aroma components, including nut, resinous, hay, green, pollen, B-sweet, pepper, H-sweet, floral, F-sweet, roasted, creamy, and Earth. Thunbergene has positively correlated with 13 aroma descriptors, such as coffee, Earth, medicinal, resinous, ester, cocoa, F-sweet, bean, incense, Hay, H-sweet, pepper, and spice. Thunbergene, also known as cembrane, is a unique compound in tobacco and an important precursor to tobacco aroma [36, 37]. It does not have an aroma in itself. It can be decomposed into solanone and diketone, which have good fragrances [49–51]. Therefore, enhancing thunbergene, mega A, and acetophenone may be used to improve the flavor of Yunnan cigars.

However, it was also found that aroma substances such as pyran and furan, neophytadiene, and phytol did not improve their self-fragrance and negatively correlated with many aroma descriptions. This may be due to interactions between aroma substances, such as synergism, compensation, and masking effects [7], or the presence of undetected component effects, or some association between detected compounds [17].

Nicotine has a significant negative correlation with 17 aroma descriptions such as Earth, H-sweet, and nut. Meanwhile, alkaloids such as nicotine and 2,3′-dipyridyl have a significant negative correlation with multiple aroma descriptions. As is well known, alkaloids such as nicotine have no odor, but they have a certain irritancy effect on the senses [27, 28]. We speculate that the high content of nicotine may lead to a strong irritancy effect impact on the smell senses that may reduce the human body's perception of fragrance. Therefore, appropriately decreasing the content of alkaloids such as nicotine in Yunnan cigars can improve their aroma.

## 4. Conclusions

In this paper, SPME–HS–GC/MS was used to analyze Yunnan cigars from four origins, and the VOCs were accurately identified through standard library marching and retention index comparison. The aroma characteristics of Yunnan cigars were studied through sensory analysis. These results were studied using PCA, LDA, and Pearson correlation analysis. The specific results obtained are as follows:

1. In Yunnan cigars, the content of nicotine and neophytadiene accounted for over 90% of the total VOCs content. Nicotine was significantly positively correlated with neophytadiene, phytol, pyran, and furan.
2. The cigars from the four origins can be classified by the PCA of VOCs. Four region discrimination functions were established through LDA of 14 compounds, and the accuracy of initial validation and leave-one-out cross-validation were both 100%.
3. Yunnan cigar sensory analysis was conducted according to standards, and results showed that the aroma characteristics of Yunnan cigars were woody, roasted, F-sweet, Bean, and scorched. Acetophenone, mega A, and thunbergene were positively correlated with multiple aroma descriptors, while nicotine was negatively correlated with various aroma descriptors. Appropriately increasing acetophenone, mega A, and thunbergene, and reducing nicotine may improve the aroma of Yunnan cigars.

## Figures and Tables

**Table 1:** VOCs in Yunnan cigars from four origins.

CAS	Chemical compound	RT calculated	RI theoretical	Class	Content (mg/kg)				Odor threshold/(mg/kg)^1^
					LC	YX	DH	PE	
000503-74-2	Butanoic acid, 3-methyl-	888	863	Acids	—	3.21 ± 3.63^a^	1.06 ± 0.9^a^	1.53 ± 1.19^a^	0.49
000105-43-1	Pentanoic acid, 3-methyl-	1005.5	—	Acids	1.62 ± 0.66^a^	—	—	—	0.28
000112-05-0	Nonanoic acid	1349.3		Acids	—	0.63 ± 0.46^b^	—	4.1 ± 1.5^a^	3.0
024070-70-0	3-Methylcyclopentyl acetate	902.4	905	Esters	—	0.24 ± 0.13^a^	—	0.27 ± 0.03^a^	—
1000309-04-5	Phthalic acid, isobutyl octyl ester	1863.4	—	Esters	0.75 ± 0.65^a^	—	—	—	—
000564-20-5	Sclareolide	2107.4	2089	Esters	1.83 ± 0.55^b^	0.99 ± 0.08^c^	2.85 ± 0.32^a^	—	—
000100-52-7	Benzaldehyde	969.1	962	Aldehyde	2.23 ± 0.82^bc^	1.83 ± 0.18^c^	3.03 ± 0.12^ab^	3.6 ± 0.96^a^	0.024
000122-78-1	Benzeneacetaldehyde	1050.9	1045	Aldehyde	0.36 ± 0.43^a^	0.26 ± 0.03^ab^	—	0.39 ± 0.07^a^	0.03
004313-03-5	2,4-Heptadienal, (E,E)-	1074.6	—	Aldehyde	—	0.55 ± 0.06^a^	—	—	0.056
000110-93-0	5-Hepten-2-one, 6-methyl-	985.6	986	Ketones	—	0.69 ± 0.04^a^	—	—	0.05
000098-86-2	Acetophenone	1073.7	1065	Ketones	0.93 ± 0.31^a^	0.23 ± 0.25^b^	—	0.12 ± 0.15^b^	0.036
030086-02-3	3,5-Octadien-2-one, (E,E)-	1096.3	1073	Ketones	—	0.37 ± 0.12^a^	—	—	0.1
001604-28-0	6-Methyl-3,5-heptadiene-2-one	1107.1	1107	Ketones	1.19 ± 0.22^a^	1.44 ± 0.14^a^	—	1.12 ± 0.38^a^	—
001125-21-9	Ketoisophorone	1151.2	1144	Ketones	—	0.39 ± 0.02^a^	—	0.27 ± 0.25^a^	0.025
017283-81-7	Dihydro-*β*-ionone	1445.5	1433	Ketones	0.86 ± 0.11^b^	—	1.28 ± 0.08^a^	—	0.01
003796-70-1	Geranylacetone	1452.3	1453	Ketones	—	6.06 ± 0.56^a^	5.81 ± 0.3^a^	4.99 ± 0.75^b^	0.06
000502-69-2	2-Pentadecanone, 6,10,14-trimethyl-	1846.4	1844	Ketones	6.83 ± 1.96^b^	—	12.84 ± 1.5^a^	17.47 ± 16.49^a^	—
001203-08-3	4-(2,6,6-Trimethylcyclohexa-1,3-dienyl)but-3-en-2-one	1487.4	1485	Ketones	—	—	—	0.8 ± 0.19^a^	—
038818-55-2	Megastigmatrienone A	1573.2	1473	Ketones	4 ± 0.79^b^	7.06 ± 3.36^a^	4.07 ± 0.36^b^	2.41 ± 0.9^b^	0.00081^2^
000141-10-6	Pseudoionone	1587.7	1581	Ketones	—	0.58 ± 0.06^a^	—	—	—
038818-55-2	Megastigmatrienone B	1591.6	1473	Ketones	18.72 ± 3.49^a^	2.23 ± 3.32^c^	18.44 ± 1.46^a^	12.81 ± 3.25^b^	0.00205^2^
038818-55-2	Megastigmatrienone	1626.4	1473	Ketones	10.98 ± 1.88^a^	—	11.7 ± 0.95^a^	—	0.00386^2^
034318-21-3	3-Oxo-*α*-ionol	1653.8	1647	Ketones	2.19 ± 1.67^a^	1.89 ± 0.45^a^	2.13 ± 0.28^a^	1.68 ± 0.31^a^	0.068
072777-88-9	(3S,5R,8S,7Z,9*ζ*)-5,6-Epoxy-7-megastigmene-3,9-diol	1678	—	Ketones	0.97 ± 0.21^b^	—	2.02 ± 0.61^a^	—	0.38
038274-01-0	3-Buten-2-one, 5,6-epoxy-3-hydroxy-*β*-ionone	1695.6	—	Ketones	0.74 ± 0.15^b^	—	1.47 ± 0.15^a^	—	—
036151-02-7	3-Oxo-7,8-dihydro-a-ionol	1713.8	1711	Ketones	3.54 ± 0.65^a^	—	—	—	—
000762-29-8	Farnesylacetone	1913.1	1921	Ketones	0.69 ± 1^b^	2.63 ± 0.62^a^	—	0.71 ± 1.24^b^	0.087
001117-52-8	E,E-Farnesylacetone	1915.8	1919	Ketones	0.63 ± 0.53^b^	0.8 ± 1.49^b^	—	3.28 ± 0.37^a^	0.087
000104-76-7	1-Hexanol, 2-ethyl-	1029.6	1030	Alcohols	—	0.37 ± 0.1^a^	—	—	0.27
000060-12-8	Phenylethyl alcohol	1117.5	1116	Alcohols	1.13 ± 0.16^a^	0.32 ± 0.06^c^	0.8 ± 0.54^b^	—	0.000015
001490-04-6	Menthol	1186	1169	Alcohols	—	1.86 ± 0.25^b^	—	2.51 ± 2.43^a^	0.1
025269-17-4	Thunbergol	2068.3	2073	Alcohols	6.61 ± 2.42^a^	—	2.47 ± 0.3^b^	—	—
000150-86-7	Phytol	2111.5	2114	Alcohols	2.06 ± 0.62^b^	1.17 ± 0.17^b^	4.17 ± 1.01^b^	7.44 ± 3.81^a^	0.64
000629-50-5	Tridecane	1300.2	1300	Hydrocarbons	—	0.46 ± 0.04^a^	—	0.4 ± 0.03^b^	42
000629-59-4	Tetradecane	1403.1	1400	Hydrocarbons	0.85 ± 0.2^a^	1.06 ± 0.12^a^	—	0.65 ± 0.62^a^	1
000544-76-3	Hexadecane	1602.9	1600	Hydrocarbons	1.02 ± 0.12^a^	0.82 ± 0.32^ab^	—	0.61 ± 0.1^b^	>13000
000629-62-9	Pentadecane	1500.7	1500	Hydrocarbons	—	0.5 ± 0.05^b^	—	0.59 ± 0.08^a^	>13000
002050-24-0	Benzene, 1,3-diethyl-5-methyl-	1626.4	—	Hydrocarbons	—	6.49 ± 0.88^b^	—	9.13 ± 2.4^a^	—
033717-93-0	1-Heptene, 2-isohexyl-6-methyl-	1730.6	—	Hydrocarbons	0.84 ± 0.61^b^	—	3.16 ± 0.35^a^	—	—
000504-96-1	Neophytadiene	1843.6	1837	Hydrocarbons	523.96 ± 87.18^b^	553.89 ± 103.3^b^	725.15 ± 71.31^b^	1107.06 ± 237.87^a^	—
001898-13-1	Thunbergene	1949.4	1939	Hydrocarbons	3.1 ± 0.85^a^	0.97 ± 0.62^b^	—	0.27 ± 0.36^bc^	—
005746-86-1	(±)-Nornicotine	1434.6	1435	Alkaloids	1.28 ± 0.38^b^	—	4.54 ± 0.94^a^	1.71 ± 0.2^b^	—
000532-12-7	Myosming	1440.9	1427	Alkaloids	17.04 ± 3.32^b^	15.23 ± 1.43^b^	35.22 ± 2.69^a^	34.7 ± 6.75^a^	—
000546-28-1	*β*-Cedrene	1447.5	1421	Alkaloids	2.26 ± 0.56^a^	—	—	—	—
000054-11-5	S-Nicotine	1369.4	1361	Alkaloids	2037.93 ± 371.12 ^d^	2855.69 ± 746.38^c^	3706 ± 175.52^b^	5666.44 ± 641.12^a^	—
000487-19-4	Nicotyrine	1488.2	1488	Alkaloids	26.76 ± 2.26^b^	33.69 ± 5.18^a^	—	38.08 ± 5.53^a^	—
040774-73-0	Anabasine	1505.9	1525	Alkaloids	2.17 ± 0.51^b^	—	—	4.86 ± 1.28^a^	—
002743-90-0	Anatabine	1535	—	Alkaloids	13.36 ± 5.37^b^	1.14 ± 0.58^c^	24.82 ± 3.23^a^	24.27 ± 4.27^a^	—
000350-03-8	Ethanone, 1-(3-pyridinyl)-	1114.4	1113	Alkaloids	1.24 ± 0.13^c^	7.97 ± 1.54^a^	4.29 ± 0.2^b^	3.78 ± 0.52^b^	—
000581-50-0	2,3′-Dipyridyl	1552.2	1556	Alkaloids	20.8 ± 2.97^c^	19.02 ± 3.48^c^	72.21 ± 8.36^a^	55.14 ± 11.77^b^	—
001008-88-4	Pyridine, 3-phenyl-	1480.9	1467	Alkaloids	0.5 ± 0.54^a^	—	—	0.52 ± 0.1^a^	—
000486-56-6	Cotinine	1717.5	1713	Alkaloids	1.6 ± 0.39^b^	1.03 ± 1.1^b^	1.81 ± 0.22^ab^	2.61 ± 0.47^a^	—
000109-08-0	Pyrazine, methyl-	826.8	831	Heterocyclics	0.84 ± 0.31^a^	0.13 ± 0.14^b^	—	0.33 ± 0.33^b^	0.06
019895-35-3	6-Ethyl-5,6-dihydro-2H-pyran-2-one	1169.4	1160	Heterocyclics	1.23 ± 1.73^b^	5.25 ± 2.96^a^	7.34 ± 0.36^a^	7.47 ± 0.97^a^	0.67
000675-20-7	2-Piperidinone	1184.4	1174	Heterocyclics	1.32 ± 0.28^a^	1.15 ± 0.74^a^	—	1.12 ± 0.42^a^	—
1000245-55-1	3-(4,8,12-Trimethyltridecyl) furan	1970.5	—	Heterocyclics	1.49 ± 0.31^a^	0.59 ± 0.64^b^	1.96 ± 0.29^a^	1.71 ± 0.39^a^	0.018
000766-39-2	2,5-Furandione, 3,4-dimethyl-	1033.2	1038	Heterocyclics	—	0.35 ± 0.06^a^	—	0.35 ± 0.08^a^	—
000872-50-4	2-Pyrrolidinone, 1-methyl-	1039.9	1044	Heterocyclics	0.45 ± 0.62^b^	1.17 ± 0.14^a^	1.28 ± 0.07^a^	1.04 ± 0.29^a^	17.11
020189-42-8	1H-Pyrrole-2,5-dione, 3-ethyl-4-methyl-	1248.7	1239	Heterocyclics	—	0.54 ± 0.22^a^	—	0.56 ± 0.1^a^	—
000077-67-8	Ethosuximide	1283.4	—	Others	2.48 ± 0.31^a^	—	—	1.78 ± 0.65^b^	—
000106-50-3	1,4-Benzenediamine	1234.7	—	Others	—	1.21 ± 0.57^a^	—	—	—
000104-66-5	Benzene, 1,1'-[1,2-ethanediylbis(oxy)]bis-	1818.4	1811	Others	1.16 ± 0.74^b^	1.5 ± 0.31^b^	2.59 ± 1.04^b^	1.29 ± 0.5^a^	—

*Note*: Values are shown as mean ± SD. The symbol “—” denotes not detected or not found.

^a–d^Significant differences in origins (*p* < 0.05).

^1^The odor threshold of most VOCs is accord to reference [24].

^2^The odor threshold is referring to reference [25].

**Table 2:** Fisher's linear discriminant classification function coefficients.

Essential variables	Origin			
	DH	LC	PE	YX
Pyrazine, methyl-	−1620.2	275.573	−1217.91	−1752.36
5-Hepten-2-one, 6-methyl-	−712.669	3211.251	−23430.4	187508.2
2,5-Furandione, 3,4-dimethyl-	6432.558	−1959.07	9705.665	−7267.86
Acetophenone	−1535.55	456.287	−2637.97	10073.46
2-Piperidinone	−388.769	154.721	−718.179	1647.953
Ethosuximide	298.343	−188.654	1179.589	−5140.89
Tridecane	−1294.68	−1502.66	13132.05	−93180.3
Megastigmatrienone	67.302	−2.61	−45.119	1164.649
3,5,9-Undecatrien-2-one, 6,10-dimethyl-	7044.042	4056.862	−36527.9	369110.1
Hexadecane	331.861	−281.055	1858.72	−8418.87
2-Cyclohexen-1-one, 4-(3-hydroxybutyl)-3,5,5-trimethyl-	−310.897	67.128	−382.149	1566.462
5,9,13-Pentadecatrien-2-one, 6,10,14-trimethyl-	119.463	100.434	−812.056	7951.105
3-(4,8,12-Trimethyltridecyl) furan	−653.946	100.676	−344.699	−3857.25
Constant	−2452.46	−191.98	−3459.42	−153140

**Table 3:** The validation of Fisher's linear discriminant classification function.

Authentication			Predicted group membership				Total
			DH	LC	PE	YX	
Original verificationa	Number	DH	4	0	0	0	4
		LC	0	8	0	0	8
		PE	0	0	7	0	7
		YX	0	0	0	8	8
	Accuracy %	DH	100.0	0.0	0.0	0.0	100.0
		LC	0.0	100.0	0.0	0.0	100.0
		PE	0.0	0.0	100.0	0.0	100.0
		YX	0.0	0.0	0.0	100.0	100.0

Cross-validationb	Number	DH	4	0	0	0	4
		LC	0	8	0	0	8
		PE	0	0	7	0	7
		YX	0	0	0	8	8
	Accuracy %	DH	100.0	0.0	0.0	0.0	100.0
		LC	0.0	100.0	0.0	0.0	100.0
		PE	0.0	0.0	100.0	0.0	100.0
		YX	0.0	0.0	0.0	100.0	100.0

^a^The total initial determination accuracy is 100%.

^b^The total cross-determination accuracy is 100%.

**Table 4:** The descriptors frequency, intensity, and GM of Yunnan cigars and its highest significance correlation compounds.

Descriptors	Frequency (%)	Intensity (%)	GM (%)	The highest significance correlation coefficient and compounds
Nut	25.31	77.78	44.37	Megastigmatrienone A(0.682⁣^∗∗^), 1-hexanol, 2-ethyl- (0.572⁣^∗∗^); cotinine(-0.685⁣^∗∗^); S-nicotine (−0.632⁣^∗∗^)
Bean	33.41	81.48	52.17	Acetophenone(0.579⁣^∗∗^), thunbergene(0.568⁣^∗∗^); 2,3′-bipyridyl(-0.629⁣^∗∗^), myosming(-0.557⁣^∗∗^)
Coffee	23.7	66.67	39.75	Thunbergene(0.761⁣^∗∗^), acetophenone(0.751⁣^∗∗^); 6-ethyl-5,6-dihydro-2H-pyran-2-one(-0.640⁣^∗∗^), geranylacetone(-0.594⁣^∗∗^)
Cocoa	17.16	40.74	26.44	3-Oxo-7,8-dihydro-a-ionol(0.614⁣^∗∗^), thunbergene (0.611⁣^∗∗^); 6-ethyl-5,6-dihydro-2H-pyran-2-one(-0.605⁣^∗∗^), geranylacetone (−0.550⁣^∗∗^)
Woody	41.07	96.3	62.89	Tetradecane (0.481⁣^∗^),6-methyl-3,5-heptadiene-2-one (0.407⁣^∗^)
Spice	19.14	70.37	36.7	Hexadecane (0.422⁣^∗^); thunbergene (0.407⁣^∗^) 6-ethyl-5,6-dihydro-2H-pyran-2-one(-0.551⁣^∗∗^); 2,3′-dipyridyl (−0.453⁣^∗^)
Fruity	8.4	18.52	12.47	Acetophenone(0.414⁣^∗^), *β*-cedrene (0.395⁣^∗^); geranylacetone (−0.391⁣^∗^)
F-sweet	35.56	77.78	52.59	Thunbergene (0.597⁣^∗∗^), tetradecane (0.570⁣^∗∗^); 2,3′-dipyridyl (−0.722⁣^∗∗^), benzaldehyde (−0.632⁣^∗∗^)
B-sweet	30.17	77.78	48.44	Megastigmatrienone a (0.528⁣^∗∗^), 1-Hexanol, 2-ethyl- (0.510⁣^∗∗^); phytol (−0.581⁣^∗∗^), myosming (−0.574⁣^∗∗^)
H-sweet	26.05	66.67	41.67	5-Hepten-2-one, 6-methyl- (0.502⁣^∗∗^), megastigmatrienone a (0.500⁣^∗∗^); myosming (−0.682⁣^∗∗^), S-nicotine (−0.642⁣^∗∗^)
Floral	20.62	48.15	31.51	2,4-Heptadienal, (E,E)-(0.594⁣^∗∗^), 5-hepten-2-one, 6-methyl- (0.572⁣^∗∗^); anatabine (−0.580⁣^∗∗^), benzaldehyde(-0.532⁣^∗∗^)
Incense	8.15	25.93	14.53	Thunbergene (0.544⁣^∗∗^), pentanoic acid, 3-methyl- (0.423⁣^∗^); 6-ethyl-5,6-dihydro-2H-pyran-2-one(-0.467⁣^∗^), S-nicotine (−0.445⁣^∗^)
Medicinal	26.11	55.56	38.09	Acetophenone (0.758⁣^∗∗^), *β*-cedrene (0.754⁣^∗∗^); geranylacetone (−0.629⁣^∗∗^), S-nicotine (−0.622⁣^∗∗^)
Creamy	15.93	29.63	21.72	2,4-Heptadienal, (E,E)- (0.535⁣^∗∗^), 5-hepten-2-one, 6-methyl- (0.530⁣^∗∗^); megastigmatrienone B (−0.533⁣^∗∗^), anatabine (−0.470⁣^∗^)
Resinous	24.26	62.96	39.08	Thunbergene (0.658⁣^∗∗^), acetophenone (0.607⁣^∗∗^); 2,3′-dipyridyl (−0.620⁣^∗∗^), S-nicotine (−0.566⁣^∗∗^)
Roasted	35.48	85.19	54.98	Megastigmatrienone a (0.416⁣^∗^), phthalic acid, isobutyloctyl ester(0.399⁣^∗^); S-nicotine (−0.395∗)
Hay	20.86	66.67	37.3	Megastigmatrienone a (0.566⁣^∗∗^), farnesylacetone (0.521⁣^∗∗^); neophytadiene (−0.666⁣^∗∗^), phytol (−0.658⁣^∗∗^)
Leather	11.85	37.04	20.95	1,4-Benzenediamine (0.383⁣^∗^)
Pepper	29.14	59.26	41.55	Megastigmatrienone a (0.506⁣^∗∗^), acetophenone (0.422⁣^∗^); S-nicotine (−0.498⁣^∗∗^), 2,3′-dipyridyl (−0.470⁣^∗^)
Earth	36.67	55.56	45.13	Thunbergene (0.701⁣^∗∗^), acetophenone (0.651⁣^∗∗^); S-nicotine (−0.776⁣^∗∗^), 6-ethyl-5,6-dihydro-2H-pyran-2-one (−0.771⁣^∗∗^)
Ester	18.15	33.33	24.6	Acetophenone (0.664⁣^∗∗^), *β*-cedrene (0.656⁣^∗∗^); geranylacetone (−0.588⁣^∗∗^), 6-ethyl-5,6-dihydro-2H-pyran-2-one (−0.569⁣^∗∗^)
Protein	17.96	70.37	35.55	Benzaldehyde (0.418⁣^∗^)
Metallic	16.54	59.26	31.31	1,4-Benzenediamine (0.403⁣^∗^), farnesylacetone (0.394⁣^∗^); anatabine (−0.385⁣^∗^)
Green	21.86	66.67	38.18	Megastigmatrienone a (0.565⁣^∗∗^), phenylethyl alcohol (0.412⁣^∗∗^); neophytadiene (−0.390⁣^∗^), S-nicotine (−0.451⁣^∗^)
Scorched	30.35	96.3	54.06	—
Pollen	4.44	18.52	9.07	Megastigmatrienone a (0.531⁣^∗∗^), 1-hexanol, 2-ethyl- (0.450⁣^∗^); 3-(4,8,12-trimethyltridecyl) furan(-0.496⁣^∗∗^), anatabine (−0.471⁣^∗^)
Ligneous	22.15	74.07	40.5	Benzaldehyde (−0.500⁣^∗∗^), 2,3′-dipyridyl (−0.439⁣^∗^)

⁣^∗^*p*≤0.05; ⁣^∗∗^*p*≤0.01.

## Data Availability

The majority of the data used to support the findings of this study are included within the article and the supporting information files. Other data are available from the corresponding author upon request.

## Supporting Information

Table S1: Aroma descriptors and its references. Table S2: Correlation analysis between aroma and volatile compounds in Yunnan cigars.
