# Supplementary material for: Characterization of Volatile Organic Compounds and Aroma Sensory Properties in Yunnan Cigar
Source: J Anal Methods Chem. 2024 Oct 16;2024:9583022. doi: 10.1155/2024/9583022 (PMC11498996; doi:10.1155/2024/9583022)
Supplement: Supporting Information — Additional supporting information can be found online in the Supporting Information Section. [file 9583022.f1.docx]

**Characterization of volatile organic compounds and aroma sensory properties in Yunan cigar**

Yuping Wu^1^, Haiyu Zhang^2,3^, Wenyuan Wang^3^, Guanghui Kong^1^, Zaiming Li^4^, Tikun Zhang^4^, Miaochang Wang^4^, Dong Yang^4^, Chengming Zhang^3^, Yongping Li^1*^, Jin Wang^3*^

^1^ Yunnan Academy of Tobacco Agricultural Science, Yunnan 653100, Yuxi, China;

^2^ College of Chemical and Environment, Yunnan Minzu University, Kunming 650500, China;

^3^ Research and Development Center, China Tobacco Yunnan Industrial Co., Ltd, Kunming 650231, China;

^4^ Puer Branch of Yunnan Tobacco Company, Yunnan, Puer 665099, China.

* Correspondence: 1303046997@qq.com ; wangjin@iccas.ac.cn

Table S1. Aroma descriptors and its references

| **Descriptors** | **references** |
| --- | --- |
| F-sweet | Yunnan flue-cured tobacco Clean Oil |
| B-sweet | Methyl cyclopentenolone |
| H-sweet | Butyl phenylacetate |
| Hay | Alfalfa Extract |
| Roasted | 2-Acetylpyridine |
| Coffee | Coffee Extract |
| Cocoa | Cocoa Extract |
| Nut | 2-Acetylpyrazine |
| Fruity | Ethyl acetoacetate |
| Floral | Dihydroisojasmone |
| Woody | Cedrol |
| Spice | cis-Anethol |
| Creamy | 2,3-Butanedione |
| Bean | 2-Pentylfuran |
| Earth | 2-Ethyl fenchol |
| Leather | 2-Isobutylquinoline |
| Pepper | Pepper Extract |
| Resinous | α-Pinene |
| Green | Leaf alcohol |
| Medicinal | Thymol |
| Incense | Guaiacol |
| Protein | Clover Solid Extract |
| Ligneous | Crosol |
| Pollen | Ambrettolide |
| Ester | Trans-2,4-Decadienal |
| Metallic | 4,5-Epoxy-(E)-2-decenal |

Table S2. Correlation analysis between aroma and volatile compounds in Yunnan cigars

|  | nut | bean | coffee | cocoa | woody | spice | fruity | F-sweet | B-sweet | H-sweet | floral | incense | medicinal | creamy | resinous | roasted | hay | leather | pepper | earth | ester | protein | metallic | green | scorched | pollen | ligneous |
| --- | --- | --- | --- | --- | --- | --- | --- | --- | --- | --- | --- | --- | --- | --- | --- | --- | --- | --- | --- | --- | --- | --- | --- | --- | --- | --- | --- |
| Pyrazine, methyl- | -0.069 | 0.420* | 0.422* | 0.472* | 0.265 | 0.189 | 0.317 | 0.334 | -0.06 | 0.116 | -0.111 | 0.224 | 0.410* | -0.007 | 0.311 | 0.164 | -0.105 | 0.038 | 0.185 | 0.476* | 0.402* | 0.058 | 0.055 | 0.191 | 0.201 | -0.128 | 0.203 |
| Butanoic acid, 3-methyl- | -0.101 | -0.193 | -0.345 | -0.348 | -0.379 | -0.212 | -0.058 | -0.238 | 0.107 | -0.06 | 0.207 | -0.191 | -0.234 | 0.264 | -0.232 | -0.055 | -0.016 | 0.295 | -0.162 | -0.263 | -0.28 | -0.03 | 0.281 | -0.091 | -0.251 | -0.04 | -0.189 |
| 3-Methylcyclopentyl acetate | -0.203 | -0.199 | -0.509** | -0.343 | -0.041 | -0.103 | -0.228 | -0.232 | 0.03 | -0.104 | 0.168 | -0.275 | -0.36 | 0.222 | -0.335 | -0.175 | -0.14 | 0.141 | -0.253 | -0.321 | -0.295 | 0.207 | 0.064 | -0.262 | 0.024 | -0.069 | -0.174 |
| Benzaldehyde | -0.540** | -0.418* | -0.257 | -0.238 | -0.133 | -0.256 | -0.223 | -0.632** | -0.291 | -0.455* | -0.532** | -0.343 | -0.501** | -0.253 | -0.462* | -0.186 | -0.627** | -0.317 | -0.322 | -0.465* | -0.404* | 0.418* | -0.129 | -0.253 | 0.084 | -0.378 | -0.500** |
| 5-Hepten-2-one, 6-methyl- | 0.520** | 0.14 | -0.082 | -0.089 | -0.02 | 0.193 | -0.046 | 0.28 | 0.503** | 0.502** | 0.572** | 0.101 | 0.044 | 0.530** | 0.188 | 0.213 | 0.464* | 0.18 | 0.207 | 0.297 | -0.056 | 0.017 | 0.251 | 0.196 | -0.123 | 0.369 | 0.124 |
| Pentanoic acid, 3-methyl- | 0.154 | 0.517** | 0.579** | 0.604** | 0.199 | 0.311 | 0.359 | 0.373 | 0.104 | 0.403* | -0.069 | 0.423* | 0.473* | -0.046 | 0.461* | 0.179 | 0.137 | 0.186 | 0.341 | 0.649** | 0.401* | 0.064 | 0.07 | 0.227 | 0.109 | -0.105 | 0.214 |
| 1-Hexanol, 2-ethyl- | 0.572** | 0.16 | -0.018 | -0.041 | 0.023 | 0.243 | -0.045 | 0.302 | 0.510** | 0.490** | 0.529** | 0.125 | 0.004 | 0.470* | 0.248 | 0.205 | 0.461* | 0.068 | 0.264 | 0.31 | -0.088 | 0.038 | 0.181 | 0.229 | -0.073 | 0.450* | 0.132 |
| 2,5-Furandione, 3,4-dimethyl- | -0.116 | -0.149 | -0.504** | -0.344 | 0.041 | -0.07 | -0.244 | -0.181 | 0.03 | -0.078 | 0.173 | -0.213 | -0.422* | 0.221 | -0.168 | -0.172 | -0.143 | 0.003 | -0.177 | -0.286 | -0.367 | 0.289 | -0.007 | -0.201 | 0.086 | 0.069 | -0.193 |
| 2-Pyrrolidinone, 1-methyl- | -0.103 | -0.347 | -0.314 | -0.396* | -0.07 | -0.343 | -0.186 | -0.228 | -0.239 | -0.373 | 0.089 | -0.359 | -0.153 | -0.026 | -0.291 | 0.011 | -0.06 | -0.178 | -0.152 | -0.455* | -0.214 | -0.119 | 0.045 | -0.076 | -0.187 | 0.216 | -0.143 |
| Benzeneacetaldehyde | -0.09 | 0.207 | -0.014 | 0.04 | 0.104 | 0.243 | 0.109 | 0.051 | 0.159 | 0.087 | -0.036 | -0.013 | -0.115 | 0.208 | 0.115 | -0.001 | -0.271 | -0.179 | -0.03 | 0.196 | -0.157 | 0.357 | 0.074 | 0.015 | 0.211 | -0.177 | -0.11 |
| Acetophenone | 0.199 | 0.579** | 0.751** | 0.596** | 0.338 | 0.329 | 0.414* | 0.514** | 0.116 | 0.318 | 0.008 | 0.409* | 0.758** | -0.059 | 0.607** | 0.389* | 0.388* | 0.08 | 0.422* | 0.651** | 0.664** | -0.112 | 0.142 | 0.397* | 0.114 | 0.298 | 0.31 |
| 2,4-Heptadienal, (E,E)- | 0.473* | 0.111 | -0.135 | -0.114 | -0.087 | 0.174 | -0.031 | 0.243 | 0.484* | 0.450* | 0.594** | 0.061 | 0.03 | 0.535** | 0.173 | 0.201 | 0.439* | 0.212 | 0.192 | 0.243 | -0.076 | -0.012 | 0.298 | 0.197 | -0.163 | 0.336 | 0.102 |
| 3,5-Octadien-2-one, (E,E)- | 0.419* | 0.12 | -0.113 | -0.112 | -0.092 | 0.13 | 0.014 | 0.201 | 0.430* | 0.417* | 0.545** | 0.003 | 0.019 | 0.499** | 0.084 | 0.158 | 0.407* | 0.312 | 0.118 | 0.219 | -0.065 | -0.024 | 0.343 | 0.137 | -0.169 | 0.261 | 0.129 |
| 6-Methyl-3,5-heptadiene-2-one | 0.238 | 0.483* | 0.191 | 0.31 | 0.407* | 0.354 | 0.115 | 0.456* | 0.249 | 0.418* | 0.279 | 0.272 | 0.189 | 0.331 | 0.467* | 0.152 | 0.218 | 0.198 | 0.294 | 0.501** | 0.219 | 0.274 | 0.091 | 0.17 | 0.224 | 0.241 | 0.26 |
| Ethanone, 1-(3-pyridinyl)- | 0.231 | -0.183 | -0.363 | -0.367 | -0.194 | -0.052 | -0.198 | -0.091 | 0.254 | 0.142 | 0.407* | -0.178 | -0.281 | 0.429* | -0.171 | 0.018 | 0.169 | 0.116 | -0.084 | -0.131 | -0.323 | 0.059 | 0.187 | -0.034 | -0.201 | 0.185 | -0.074 |
| Phenylethyl Alcohol | 0.187 | 0.312 | 0.691** | 0.425* | 0.03 | 0.234 | 0.303 | 0.379 | -0.027 | 0.256 | 0.019 | 0.291 | 0.638** | -0.01 | 0.408* | 0.27 | 0.361 | 0.074 | 0.299 | 0.499** | 0.523** | -0.344 | 0.189 | 0.412* | -0.206 | 0.08 | 0.147 |
| Ketoisophorone | 0.118 | -0.032 | -0.397* | -0.22 | 0.139 | 0.018 | -0.181 | 0.09 | 0.087 | 0.031 | 0.342 | -0.114 | -0.26 | 0.297 | -0.015 | -0.077 | -0.024 | -0.045 | -0.026 | -0.101 | -0.244 | 0.137 | -0.028 | -0.062 | 0.081 | 0.168 | 0.021 |
| 6-Ethyl-5,6-dihydro-2H-pyran-2-one | -0.376 | -0.557** | -0.640** | -0.605** | -0.184 | -0.551** | -0.234 | -0.540** | -0.31 | -0.575** | -0.157 | -0.467* | -0.570** | -0.146 | -0.419* | -0.242 | -0.382* | -0.132 | -0.277 | -0.771** | -0.569** | 0.088 | 0.077 | -0.178 | -0.065 | -0.097 | -0.411* |
| 1,4-Benzenediamine | 0.368 | 0.116 | -0.144 | -0.144 | -0.099 | 0.098 | 0.027 | 0.19 | 0.422* | 0.394* | 0.564** | -0.007 | 0.043 | 0.529** | 0.067 | 0.183 | 0.400* | 0.383* | 0.094 | 0.187 | -0.056 | -0.024 | 0.403* | 0.142 | -0.159 | 0.205 | 0.13 |
| 2-Piperidinone | -0.132 | 0.303 | 0.097 | 0.149 | 0.159 | 0.064 | 0.243 | 0.244 | 0.003 | 0.138 | 0.139 | 0.174 | 0.198 | 0.05 | 0.456* | 0.003 | 0.151 | 0.309 | 0.165 | 0.174 | 0.13 | 0.071 | 0.19 | 0.064 | 0.06 | 0.048 | -0.005 |
| Menthol | -0.215 | -0.162 | -0.283 | -0.307 | 0.027 | 0.044 | -0.195 | -0.239 | -0.031 | 0.017 | -0.025 | -0.167 | -0.358 | 0.048 | -0.174 | 0.024 | -0.163 | 0.001 | -0.193 | -0.256 | -0.293 | 0.348 | 0.076 | -0.059 | -0.026 | 0.041 | -0.218 |
| 1H-Pyrrole-2,5-dione, 3-ethyl-4-methyl- | -0.168 | -0.185 | -0.474* | -0.36 | -0.072 | -0.13 | -0.197 | -0.238 | 0.067 | -0.099 | 0.154 | -0.246 | -0.422* | 0.234 | -0.273 | -0.163 | -0.119 | 0.141 | -0.206 | -0.327 | -0.38 | 0.234 | 0.091 | -0.215 | 0.021 | 0.014 | -0.211 |
| Nonanoic acid | -0.549** | -0.247 | -0.482* | -0.305 | 0.076 | -0.178 | -0.231 | -0.465* | -0.327 | -0.416* | -0.278 | -0.327 | -0.492** | -0.212 | -0.322 | -0.263 | -0.520** | -0.11 | -0.366 | -0.542** | -0.346 | 0.373 | -0.133 | -0.3 | 0.219 | -0.233 | -0.365 |
| Ethosuximide | -0.315 | 0.244 | 0.424* | 0.444* | 0.306 | 0.047 | 0.22 | 0.212 | -0.176 | -0.074 | -0.297 | 0.161 | 0.33 | -0.274 | 0.189 | 0.074 | -0.142 | 0.003 | 0.149 | 0.226 | 0.365 | 0.044 | -0.091 | 0.058 | 0.258 | -0.163 | 0.049 |
| Tridecane | 0.035 | -0.118 | -0.428* | -0.308 | 0.073 | 0.011 | -0.234 | -0.082 | 0.164 | 0.023 | 0.241 | -0.171 | -0.404* | 0.277 | -0.152 | -0.118 | -0.024 | 0.001 | -0.084 | -0.207 | -0.366 | 0.262 | 0.013 | -0.138 | 0.078 | 0.173 | -0.103 |
| S-Nicotine | -0.632** | -0.534** | -0.565** | -0.459* | -0.07 | -0.372 | -0.325 | -0.620** | -0.446* | -0.642** | -0.362 | -0.445* | -0.622** | -0.244 | -0.566** | -0.395* | -0.593** | -0.179 | -0.498** | -0.776** | -0.450* | 0.251 | -0.235 | -0.451* | 0.049 | -0.299 | -0.417* |
| Tetradecane | 0.279 | 0.452* | 0.216 | 0.244 | 0.481* | 0.297 | 0.118 | 0.570** | 0.12 | 0.304 | 0.355 | 0.191 | 0.348 | 0.291 | 0.506** | 0.182 | 0.272 | 0.007 | 0.32 | 0.455* | 0.275 | 0.08 | 0.074 | 0.184 | 0.125 | 0.308 | 0.365 |
| (±)-Nornicotine | -0.327 | -0.433* | -0.141 | -0.249 | -0.243 | -0.384* | -0.149 | -0.484* | -0.475* | -0.530** | -0.470* | -0.306 | -0.267 | -0.390* | -0.436* | -0.21 | -0.441* | -0.33 | -0.355 | -0.512** | -0.219 | -0.135 | -0.234 | -0.176 | -0.106 | -0.299 | -0.315 |
| Myosming | -0.559** | -0.557** | -0.417* | -0.389* | -0.027 | -0.422* | -0.318 | -0.586** | -0.574** | -0.682** | -0.493** | -0.395* | -0.537** | -0.420* | -0.498** | -0.327 | -0.612** | -0.339 | -0.439* | -0.749** | -0.385* | 0.163 | -0.336 | -0.355 | 0.039 | -0.311 | -0.358 |
| Dihydro-β-ionone | 0.03 | 0 | 0.359 | 0.186 | -0.144 | -0.061 | 0.173 | -0.033 | -0.155 | -0.064 | -0.229 | 0.106 | 0.291 | -0.214 | 0.049 | 0.128 | 0.003 | -0.062 | 0.035 | 0.122 | 0.226 | -0.217 | 0.006 | 0.142 | -0.155 | -0.13 | 0.068 |
| β-Cedrene | 0.065 | 0.546** | 0.708** | 0.591** | 0.278 | 0.262 | 0.395* | 0.447* | -0.005 | 0.222 | -0.055 | 0.311 | 0.754** | -0.072 | 0.518** | 0.337 | 0.239 | 0.075 | 0.349 | 0.608** | 0.656** | -0.12 | 0.156 | 0.319 | 0.086 | 0.057 | 0.3 |
| Geranylacetone | -0.012 | -0.503** | -0.594** | -0.550** | -0.222 | -0.273 | -0.391* | -0.412* | -0.014 | -0.219 | 0.079 | -0.351 | -0.629** | 0.129 | -0.428* | -0.197 | -0.194 | -0.207 | -0.261 | -0.518** | -0.588** | 0.144 | -0.088 | -0.208 | -0.124 | 0.053 | -0.307 |
| Pyridine, 3-phenyl- | -0.225 | 0.118 | 0.025 | 0.166 | 0.056 | 0.13 | -0.113 | -0.005 | -0.078 | 0.086 | -0.219 | 0.127 | -0.176 | -0.202 | 0.101 | -0.209 | -0.142 | 0.157 | -0.038 | 0.013 | -0.022 | 0.217 | -0.256 | -0.238 | 0.233 | -0.337 | -0.018 |
| 4-(2,6,6-Trimethylcyclohexa-1,3-dienyl)but-3-en-2-one | -0.605** | -0.328 | -0.419* | -0.264 | 0.075 | -0.228 | -0.247 | -0.477* | -0.353 | -0.498** | -0.394* | -0.319 | -0.502** | -0.306 | -0.384* | -0.334 | -0.547** | -0.195 | -0.343 | -0.572** | -0.341 | 0.34 | -0.24 | -0.374 | 0.238 | -0.257 | -0.384* |
| Pentadecane | -0.132 | -0.163 | -0.515** | -0.311 | 0.121 | -0.042 | -0.264 | -0.19 | 0.004 | -0.122 | 0.11 | -0.263 | -0.453* | 0.131 | -0.218 | -0.207 | -0.195 | -0.052 | -0.179 | -0.316 | -0.36 | 0.323 | -0.065 | -0.255 | 0.125 | 0.033 | -0.176 |
| Nicotyrine | -0.079 | 0.257 | -0.039 | 0.114 | 0.379 | 0.18 | 0.035 | 0.201 | 0.067 | 0.143 | 0.095 | 0.033 | -0.012 | 0.115 | 0.211 | -0.028 | -0.015 | 0.091 | 0.087 | 0.164 | 0.051 | 0.312 | 0.036 | -0.028 | 0.252 | 0.089 | 0.036 |
| Anabasine | -0.565** | -0.098 | -0.151 | 0.017 | 0.337 | -0.134 | -0.089 | -0.138 | -0.456* | -0.461* | -0.395* | -0.121 | -0.211 | -0.355 | -0.094 | -0.347 | -0.476* | -0.169 | -0.185 | -0.32 | -0.046 | 0.246 | -0.341 | -0.344 | 0.322 | -0.273 | -0.117 |
| Anatabine | -0.589** | -0.440* | -0.219 | -0.175 | -0.07 | -0.307 | -0.235 | -0.526** | -0.539** | -0.572** | -0.580** | -0.231 | -0.423* | -0.470* | -0.408* | -0.346 | -0.583** | -0.193 | -0.404* | -0.597** | -0.227 | 0.118 | -0.385* | -0.339 | 0.03 | -0.471* | -0.336 |
| 2,3'-Dipyridyl | -0.505** | -0.629** | -0.441* | -0.434* | -0.247 | -0.453* | -0.327 | -0.722** | -0.448* | -0.614** | -0.501** | -0.428* | -0.588** | -0.371 | -0.620** | -0.271 | -0.597** | -0.278 | -0.470* | -0.749** | -0.457* | 0.152 | -0.239 | -0.326 | -0.058 | -0.381* | -0.439* |
| Megastigmatrienone A | 0.682** | 0.317 | 0.17 | 0.033 | 0.29 | 0.186 | 0.134 | 0.491** | 0.528** | 0.500** | 0.497** | 0.271 | 0.167 | 0.405* | 0.571** | 0.416* | 0.566** | 0.103 | 0.506** | 0.393* | -0.022 | 0.074 | 0.375 | 0.565** | 0.158 | 0.531** | 0.265 |
| Pseudoionone | 0.483* | 0.124 | -0.099 | -0.104 | -0.068 | 0.161 | -0.016 | 0.242 | 0.473* | 0.458* | 0.558** | 0.06 | 0.013 | 0.513** | 0.155 | 0.178 | 0.438* | 0.222 | 0.182 | 0.256 | -0.084 | -0.007 | 0.291 | 0.183 | -0.15 | 0.344 | 0.118 |
| Megastigmatrienone B | -0.386* | -0.088 | 0.215 | 0.211 | 0.036 | -0.155 | 0.105 | -0.149 | -0.536** | -0.389* | -0.494** | -0.014 | 0.221 | -0.533** | -0.012 | -0.102 | -0.327 | -0.214 | -0.097 | -0.105 | 0.277 | -0.135 | -0.243 | -0.085 | -0.057 | -0.279 | -0.063 |
| Hexadecane | 0.165 | 0.516** | 0.303 | 0.549** | 0.299 | 0.422* | 0.258 | 0.449* | 0.24 | 0.467* | 0.224 | 0.213 | 0.591** | 0.062 | 0.470* | 0.234 | 0.277 | 0.217 | 0.351 | 0.634** | 0.588** | 0.017 | 0.19 | 0.221 | 0.032 | -0.002 | 0.186 |
| Benzene, 1,3-diethyl-5-methyl- | -0.186 | -0.194 | -0.493** | -0.317 | 0.176 | -0.1 | -0.259 | -0.17 | -0.075 | -0.187 | 0.027 | -0.24 | -0.497** | 0.055 | -0.19 | -0.217 | -0.272 | -0.138 | -0.159 | -0.355 | -0.403* | 0.331 | -0.122 | -0.22 | 0.184 | 0.045 | -0.192 |
| Megastigmatrienone | 0.047 | 0.131 | 0.489** | 0.309 | -0.035 | 0.015 | 0.231 | 0.112 | -0.143 | 0.008 | -0.184 | 0.199 | 0.443* | -0.218 | 0.193 | 0.161 | 0.117 | 0.023 | 0.109 | 0.232 | 0.382* | -0.257 | 0.003 | 0.175 | -0.124 | -0.085 | 0.161 |
| 3-Oxo-α-ionol | -0.041 | 0.104 | 0.278 | 0.105 | 0.125 | -0.113 | 0.119 | 0.07 | -0.193 | -0.151 | 0.077 | -0.1 | 0.497** | -0.114 | 0.141 | 0.238 | 0.258 | 0.057 | 0.07 | 0.012 | 0.367 | -0.199 | 0.155 | 0.07 | -0.138 | 0.205 | 0.089 |
| (3S,5R,8S,7Z,9ζ)-5,6-Epoxy-7-megastigmene-3,9-diol | 0.044 | -0.11 | 0.256 | 0.079 | -0.171 | -0.159 | 0.026 | -0.108 | -0.179 | -0.096 | -0.265 | 0.015 | 0.093 | -0.193 | -0.095 | 0.028 | -0.088 | -0.09 | -0.09 | -0.02 | 0.098 | -0.227 | -0.127 | 0.071 | -0.153 | -0.206 | -0.022 |
| 3-Buten-2-one, 4-(4-hydroxy-2,2,6-trimethyl-7-oxabicyclo[40.10.0]hept-1-yl)- | 0.012 | -0.114 | 0.252 | 0.097 | -0.193 | -0.127 | 0.062 | -0.135 | -0.179 | -0.116 | -0.251 | 0.011 | 0.154 | -0.199 | -0.064 | 0.079 | -0.073 | -0.124 | -0.024 | 0.012 | 0.112 | -0.193 | -0.034 | 0.102 | -0.187 | -0.199 | -0.013 |
| 3-Oxo-7,8-dihydro-a-ionol | 0.135 | 0.549** | 0.668** | 0.614** | 0.261 | 0.294 | 0.342 | 0.444* | 0.058 | 0.328 | -0.034 | 0.36 | 0.652** | -0.065 | 0.535** | 0.278 | 0.236 | 0.138 | 0.379 | 0.647** | 0.559** | -0.028 | 0.108 | 0.267 | 0.108 | -0.025 | 0.293 |
| Cotinine | -0.685** | -0.355 | -0.244 | -0.224 | 0.007 | -0.346 | -0.139 | -0.392* | -0.531** | -0.554** | -0.347 | -0.346 | -0.24 | -0.247 | -0.387* | -0.263 | -0.467* | 0.024 | -0.379 | -0.539** | -0.128 | 0.114 | -0.081 | -0.347 | -0.022 | -0.444* | -0.238 |
| 1-Heptene, 2-isohexyl-6-methyl- | -0.028 | -0.284 | 0.069 | -0.08 | -0.237 | -0.279 | 0.012 | -0.266 | -0.264 | -0.268 | -0.23 | -0.092 | 0.06 | -0.271 | -0.203 | 0.03 | -0.08 | -0.153 | -0.097 | -0.201 | -0.007 | -0.225 | -0.036 | 0.026 | -0.223 | -0.079 | -0.07 |
| Benzene, 1,1'-[1,2-ethanediylbis(oxy)]bis- | -0.1 | -0.231 | 0.069 | -0.224 | -0.188 | -0.161 | -0.353 | -0.171 | -0.096 | -0.172 | -0.116 | -0.293 | -0.173 | 0.117 | -0.196 | 0.107 | -0.197 | -0.348 | -0.11 | -0.234 | -0.141 | -0.121 | -0.041 | 0.059 | -0.32 | -0.227 | -0.315 |
| Neophytadiene | -0.571** | -0.416* | -0.514** | -0.342 | 0.093 | -0.313 | -0.272 | -0.514** | -0.486* | -0.566** | -0.399* | -0.364 | -0.598** | -0.278 | -0.429* | -0.376 | -0.666** | -0.223 | -0.407* | -0.629** | -0.436* | 0.376 | -0.262 | -0.390* | 0.166 | -0.345 | -0.321 |
| 2-Pentadecanone, 6,10,14-trimethyl- | -0.392* | -0.222 | -0.307 | -0.122 | 0.169 | -0.255 | -0.145 | -0.254 | -0.524** | -0.542** | -0.346 | -0.237 | -0.296 | -0.328 | -0.241 | -0.297 | -0.591** | -0.345 | -0.254 | -0.410* | -0.188 | 0.112 | -0.336 | -0.238 | 0.206 | -0.282 | -0.106 |
| Phthalic acid, isobutyl octyl ester | 0.061 | 0.463* | 0.550** | 0.506** | 0.274 | 0.112 | 0.35 | 0.265 | -0.045 | 0.128 | -0.103 | 0.107 | 0.651** | -0.083 | 0.359 | 0.399* | 0.042 | -0.142 | 0.396* | 0.562** | 0.449* | 0.081 | 0.27 | 0.333 | 0.134 | 0.082 | 0.13 |
| Farnesylacetone | 0.238 | 0.085 | 0.11 | -0.06 | 0.083 | 0.056 | 0.066 | 0.26 | 0.333 | 0.328 | 0.448* | 0.001 | 0.221 | 0.344 | 0.123 | 0.198 | 0.521** | 0.238 | 0.185 | 0.214 | 0.088 | -0.045 | 0.394* | 0.176 | 0.001 | 0.363 | 0.138 |
| E,E-Farnesylacetone | -0.365 | -0.191 | -0.399* | -0.124 | 0.16 | 0.044 | -0.261 | -0.213 | -0.111 | -0.25 | -0.117 | -0.194 | -0.347 | -0.095 | -0.205 | -0.166 | -0.389* | -0.177 | -0.166 | -0.335 | -0.201 | 0.336 | -0.218 | -0.237 | 0.225 | -0.224 | -0.229 |
| Thunbergene | 0.281 | 0.568** | 0.761** | 0.611** | 0.329 | 0.407* | 0.356 | 0.597** | 0.223 | 0.475* | 0.106 | 0.544** | 0.667** | 0.061 | 0.658** | 0.283 | 0.481* | 0.247 | 0.408* | 0.701** | 0.620** | -0.108 | 0.061 | 0.348 | 0.073 | 0.187 | 0.378 |
| 3-(4,8,12-Trimethyltridecyl) furan | -0.536** | -0.288 | -0.153 | -0.112 | -0.175 | -0.249 | -0.075 | -0.509** | -0.465* | -0.440* | -0.449* | -0.182 | -0.228 | -0.338 | -0.322 | -0.231 | -0.459* | 0.055 | -0.400* | -0.432* | -0.104 | 0.108 | -0.145 | -0.27 | -0.043 | -0.496** | -0.244 |
| Thunbergol | 0.085 | 0.373 | 0.645** | 0.503** | 0.196 | 0.189 | 0.289 | 0.383* | -0.059 | 0.172 | -0.046 | 0.348 | 0.641** | -0.153 | 0.457* | 0.196 | 0.308 | 0.184 | 0.249 | 0.427* | 0.600** | -0.244 | -0.001 | 0.206 | -0.033 | -0.011 | 0.313 |
| Sclareolide | 0.259 | 0.043 | 0.383* | 0.173 | -0.118 | -0.038 | 0.129 | 0.098 | -0.002 | 0.109 | 0.024 | 0.125 | 0.389* | -0.032 | 0.145 | 0.236 | 0.263 | 0.039 | 0.138 | 0.216 | 0.271 | -0.283 | 0.098 | 0.228 | -0.255 | 0.016 | 0.152 |
| Phytol | -0.547** | -0.356 | -0.404* | -0.255 | 0.173 | -0.324 | -0.239 | -0.388* | -0.581** | -0.595** | -0.420* | -0.341 | -0.452* | -0.347 | -0.34 | -0.336 | -0.658** | -0.34 | -0.341 | -0.559** | -0.314 | 0.27 | -0.332 | -0.352 | 0.18 | -0.308 | -0.266 |

* P≤0.05；* *P≤0.01.
